# The Spread of the Lengthening Time Effect of Emotions in Memory: A Test in the Setting of the Central Tendency Effect

**DOI:** 10.3389/fpsyg.2021.774392

**Published:** 2021-11-18

**Authors:** Sylvie Droit-Volet, Sandrine Gil

**Affiliations:** ^1^Université Clermont Auvergne, CNRS, Laboratoire de Psychologie Sociale et COgnitive (LAPSCO), Clermont-Ferrand, France; ^2^Université de Poitiers, CNRS, Centre de Recherche sur la Cognition et l’Apprentissage (CeRCA), Poitiers, France

**Keywords:** time, emotion, memory, context, central tendency effect

## Abstract

The aim of the present study was to test how the perception of an emotional stimulus colors the temporal context of judgment and modifies the participant’s perception of the current neutral duration. Participants were given two ready-set-go tasks consisting of a distribution of short (0.5–0.9 s) or long sample intervals (0.9–1.3 s) with an overlapping 0.9-s interval. Additional intervals were introduced in the temporal distribution. These were neutral for the two temporal tasks in a control condition and emotional for the short, but not the long temporal task in an emotion condition. The results indicated a replication of a kind of Vierordt’s law in the control condition, i.e., the temporal judgment toward the mean of the distribution of sample intervals (central tendency effect). However, there was a shift in the central tendency effect in the emotion condition indicating a general bias in the form of an overestimation of current intervals linked to the presence of a few emotional stimuli among the previous intervals. This finding is entirely consistent with timing mechanisms driven by prior duration context, particularly experience of prior emotional duration.

## Introduction

For decades, the number of studies devoted to time and emotion has been constantly growing. Most of these have examined the perception of the duration of emotional stimuli (facial expressions, emotional pictures, or sounds) and their immediate effect on time judgment (Droit-Volet and Meck, 2007; Lake, 2016; Droit-Volet, 2019). Although time distortions embedded in an emotional phenomenon are complex, investigators have observed that threatening stimuli produce an increase in estimated durations. This lengthening effect is thought to result from the arousal dimension of significant stimuli, which in turn accelerates the internal clock system during the measurement of time. When the clock system runs faster, more time units are produced and the current stimulus duration is judged longer. Transient inhibition or activation of dopaminergic neurons would explain the lengthening or shortening of time estimates (Cheng et al., 2016; Soares et al., 2016). These studies have investigated the immediate effect of emotion on time judgment, but not how the prior temporal experience of an emotional stimulus influences the time measurement of other encountered stimuli. Naturally, some experiments have been conducted on the temporal memory of emotional events (Cocenas-Silva et al., 2012, 2013). However, these works have addressed the memory retention of the duration of emotional stimuli, but not the dependence of the current temporal judgment on knowledge of past duration events with a certain emotional color. The aim of the present study was therefore to test how the perception of an emotional stimulus colors the temporal context of judgment and modifies the participant’s decision on the current stimulus duration.

According to the Bayesian theoretical approach, the human mind processes the properties of a stimulus in combination with those of previously processed information. Our perception of the world is considered to be endlessly modulated and optimized by inferences derived from previous experience with it. The mind is considered as a “Bayesian optimizer” (e.g., Tenenbaum et al., 2011). In the time perception domain, a Bayesian perceiver does not judge a stimulus duration (measured by an internal clock) solely on the basis of its mere isolated value (which would be constant across successive trials). He/she produces a subjective estimate (posterior) that results from the currently perceived stimulus (likelihood) weighted with the prior experience (prior) (Shi et al., 2013; Freestone and Church, 2016). The influence of prior temporal distribution on time judgment was observed many years ago by Karl von Vierordt (1868) in his studies using the reproduction task (Fortin and Rousseau, 1998; Lejeune and Wearden, 2009; Bausenhart et al., 2016). In this task, a participant is given a series of trials with different target durations (e.g., from 1 to 7 s), with a single duration being presented and reproduced per trial. Vierordt’s studies showed that shorter durations are reproduced as longer than they really are, whereas longer durations are reproduced as shorter. This typical result, replicated in numerous experiments, is now known as Vierordt’s law. This law accounts for the outcome of temporal judgment toward the mean of the distribution of sample durations, and illustrates the effect of knowledge (priors) in the measurement of current time. Broadly speaking, evidence shows that this result observed on the temporal dimension of the processing of information is common to the whole of our sensory system, consistently with the *central tendency effect* (Hollingworth, 1910; for a review see Glasauer and Shi, 2021).

More recently, Jazayeri and Shadlen (2010) developed a paradigm that makes it possible to further examine the effect of temporal context on time judgment in a reproduction task called the ready-set-go task (see section “Materials and Methods”). As in all reproduction tasks, participants have to reproduce temporal intervals, with a sample interval being proposed in each trial. The originality of their paradigm lies in the fact that participants are given two separate tasks with different distributions of sample intervals, one with short intervals ranging from 494 to 847 ms and the other with long intervals from 847 to 1,200 ms. Crucially, one sample interval (847-ms interval) overlaps the two distributions. According to certain results, the same overlapping interval appeared to be estimated shorter when presented with short sample intervals than with long sample intervals. This confirms the prior-dependent bias and suggests that participants adopt a “Bayesian strategy to reproduce time intervals” (Jazayeri and Shadlen, 2010, p. 1021). This paradigm has since been used in other studies that have found similar results (e.g., Karaminis et al., 2016; Hallez et al., 2018; Zimmermann and Cicchini, 2020).

The original aim of the present study was to test the role of prior emotional durations on present time judgment. We therefore used Jazayeri and Shadlen’s paradigm and introduced certain intervals in the form of high-arousal emotional stimuli (i.e., facial expressions, one of the most emotional stimuli used in the literature) into the distribution of sample intervals. In this context, it was necessary to exclude the possibility of emotional reactions triggered during the emotion interval, rather than knowledge of the intervals themselves, from affecting the judgment of subsequent sample intervals. First, it is easy to observe that previous studies on timing of emotional stimuli have randomly alternated the presentation of neutral and emotionally charged stimuli and have nevertheless revealed a significant difference in the estimated durations of the two types of stimuli. At the experimental level, this suggests that there is no emotional contagion *per se* within the trials performed (Hess et al., 1998). Second, by way of an additional precaution, we used a long interval between two trials ranging from 4 to 6 s. Indeed, it has been demonstrated that the effect of an emotional picture on time estimates (lengthening of time) is no longer observed after 2 s (for a review, see Droit-Volet, 2019). This therefore ensured that the emotional reaction to a stimulus was restricted to the corresponding trial.

In summary, the lengthening effect of isolated emotional stimuli compared to neutral stimuli on time judgments has been widely demonstrated. In Jazayeri and Shadlen’s paradigm, the introduction into the distribution of sample intervals of intervals associated with an angry facial expression that induces a temporal overestimation could therefore change the temporal context, thus shifting the mean of the temporal distribution in memory toward a longer value. Since the perception of the current duration is thought to be weighted by prior experience, the duration of the current interval would be judged to be longer with emotional than neutral priors. If this hypothesis was correct, we predicted that when the emotional intervals are introduced into the distribution of short sample intervals (short task) and not into that of long intervals (long task), a lengthening bias will be observed in the short condition, with the result that the “Vierordt effect” will be modified. In particular, in Jazayeri and Shadlen’s study, the sample interval (0.9-s) common to the two temporal distributions (short and long) was judged shorter in the short than in the long interval distribution (central tendency effect). In the present study, with the inclusion of an emotional context that would produce an overestimation bias, the overlapping interval should not be judged shorter even if it is part of a short temporal distribution. The central tendency effect depending on the duration range would therefore disappear in favor of an emotional context effect, in contrast to a non-emotion condition in which only neutral intervals were presented in both distributions.

## Materials and Methods

### Participants

The final sample consisted of 80 participants (mean age = 19.78, SD = 2.89). Two additional participants were excluded from this sample because they did not understand the instructions and considered the task to be a reaction time task. The participants were first and second-year psychology students at the French Clermont Auvergne University (UCA) who participated in the study in return for course credits. They signed a consent form that was approved by the UCA Research Ethics Committee (IRB00011540-2019-32).

### Material

**FIGURE 1:**
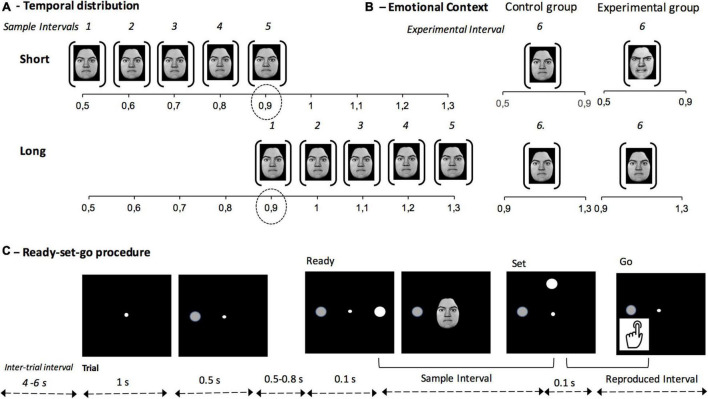
Experimental design of the emotion-modified version of the ready-set-go procedure. **(A)** Temporal distribution description according to the Short vs. Long condition. The interval of 0.9 s is circle in dotted line because it is common to both temporal distributions. **(B)** Operationalized Emotional Context as a function of temporal distribution (Short vs. Long) and group condition [Control vs. Experimental (i.e., Emotion)]. **(C)** Detailed description of the ready-set-go general task, with a trial example.

The participants responded alone in a room in our laboratory in front of a computer. The events presented on the computer were programmed using e-prime software. The facial expressions used were the faces of three different women expressing either neutrality or anger. These faces were in black and white and came from Ekman and Friesen (1976) Pictures of Facial Affect (Figure 1).

### Procedure

The procedure used was an emotion-based version of the ready-set-go procedure used by Jazayeri and Shadlen (2010). As illustrated in Figure 1, in this task, the participants were instructed to look at a white dot in the center of the computer screen and to maintain their visual fixation throughout the trial. A small gray circle then became visible on the left. After a delay randomly chosen between 0.25 and 0.85 s, two circles (ready and set) were presented successively for 100 ms, separated by a sample temporal interval. The participants had to immediately reproduce this sample interval. Successive trials were separated by an inter-trial interval going from 4 to 6 s. The reproduced interval was therefore the duration between the set cue and the participant’s key-press. In our emotion-based version of this procedure, a neutral facial expression was always presented during the sample intervals, except for the additional “emotional” intervals, which were either neutral or emotional as a function of the emotion condition (control vs. emotion).

The participants were given two successive ready-set-go tasks (Short vs. Long): one with the distribution of short sample intervals, and the other with the distribution of long sample intervals. The task-order was counterbalanced between subjects. Three trial demonstrations were given at the beginning of each task. One sample interval overlapped these two temporal distributions. The same interval duration of 0.9 s was thus included in the two different temporal contexts. For the Short task, the five sample intervals to be reproduced were 0.5, 0.6, 0.7, 0.8, and 0.9 s, and for the Long task 0.9, 1.0, 1.1, 1.2, and 1.3 s. A sixth emotional interval was added. The duration of this emotional interval was randomly chosen between 0.5 and 0.9 s for the Short task and between 0.9 and 1.3 s for the Long task. Each task was composed of 54 trials, i.e., nine blocks of six trials: the five sample intervals and the emotional interval. The trial order was random within each trial block. This made a proportion of emotional intervals of 0.16 per task.

The participants were arbitrarily assigned to either the control group or the emotion group. For the control group, the neutral faces were presented for the emotional intervals in both the Short and the Long task. For the emotion group, the angry faces were presented for the emotional intervals in the Short task but not in the Long task. Therefore, only the emotional context, i.e., presence of an angry or neutral face for the emotional intervals, changed between the groups in the Short ready-set-go task. The neutral face and the angry face were randomly taken from a set of three different faces.

## Results

**FIGURE 2:**
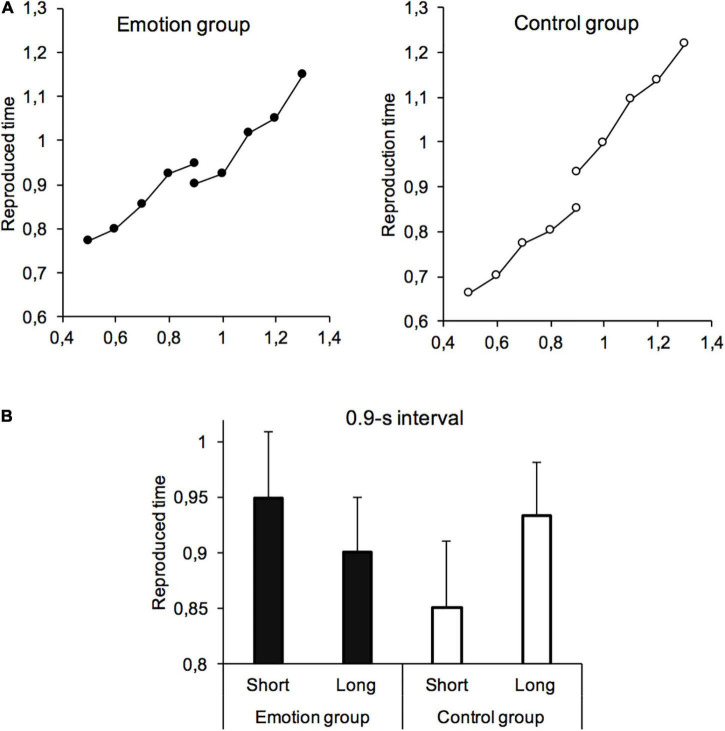
Reproduced interval. **(A)** Reproduced time (seconds) plotted against the sample intervals for the temporal task with the distribution of short intervals (Short) and that with the distribution of long intervals (Long) for the control group and the emotion group. **(B)** Reproduced interval for the 0.9-s overlapping interval for the Short and the Long temporal task.

Figure 2 shows the reproduced intervals for the different sample intervals (all with stimulus durations in the form of faces) in the emotion and the control group for the Short and the Long task. As observed in all reproduction tasks, the curve of reproduced intervals increased with the duration of the target intervals, and this in all conditions tested. More interestingly, in the control group, a kind of Vierordt-related effect was replicated with our new version of the ready-set-go procedure. The ANOVA conducted on the time estimates for the overlapping interval (0.9 s) with task (Short vs. Long) as within-subject factor and task-order as between-subjects factor showed a significant main effect of the task, *F*(1,38) = 6.03, *p* = 0.01, η^2^*_*p*_* = 0.15. The order effect and the order × task interaction were not significant (*p* > 0.10). Therefore, the same sample interval (0.9 s) was judged shorter when included in a temporal context with shorter (*M* = 0.851, SD = 0.30) rather than longer (*M* = 0.933, SD = 0.287) sample intervals.

By contrast, for the emotion group, the ANOVA showed a significant order × task interaction for the overlapping interval, *F*(1,38) = 5.96, *p* = 0.02, η^2^*_*p*_* = 0.14. When the Short task was performed first, there was no difference in the 0.90-s estimates between the Short (*M* = 0.826, SD = 0.304) and the Long task (*M* = 0.820, SD = 0.243), *t*(20) = −0.64, *p* = 0.53, Cohen’s *d* = −0.14. However, when the Long task was performed first, the overlapping interval was judged longer, at a significant level, in the Short task (*M* = 1114, SD = 1188) than in the Long task (*M* = 0.989, SD = 0.872), *t*(18) = 2.44, *p* = 0.025, Cohen’s *d* = 0.56. There was therefore a reversal of the time curves in this emotion condition, with the overlapping interval judged longer in the short task than in the long task.

To better understand this general bias in the judgment of short intervals in the emotion group, we calculated the slope and the intercept parameters of temporal curves from the linear regression performed on the averaged data (curves Figure 2). Table 1 presents these parameters. It appears differences between the emotion and the control condition for the intercept values rather than for the slope-values in the Short task. This indicates a general overestimation of time estimates in the short intervals for the emotion group. The overestimation of the overlapping interval (0.9 s) in the emotion condition, despite being included in a short interval distribution, would therefore reflect a shift in the memory representation of this temporal distribution when samples were associated with emotional stimuli.

**TABLE 1:** Slope and intercept obtained from the linear regression performed on the averaged data in each condition.

	Slope	Intercept^1^	Predicted value at the central time^2^	*R* ^2^	*p* value
**Control group**					
Short	0.478	422.98	757.46	0.99	*0*.*0007*
Long	0.716	289.4	1076.58	0.99	*0*.*0004*
**Emotion group**					
Short	0.476	526.6	860.03	0.98	*0*.*0015*
Long	0.626	320	1008.94	0.96	*0*.*0029*
**Emotion group – Long first**					
Short	0.502	651.26	1002.7	0.99	*0*.*001*
Long	0.796	232.86	1109.06	0.97	*0*.*007*
**Emotion group – Short first**					
Short	0.453	413.84	730.939	0.93	*0*.*02*
Long	0.472	398.75	918.356	0.98	*0*.*004*

*^1^Intercept in ms; ^2^Predicted value for 700 ms (Short) or 1,100 ms (long).*

Further statistical analyses in the Short task for the emotion group were performed using a mixed linear model with the reproduced interval as dependent variable and the participants as random effect. A first analysis with the emotion as fixed factor confirmed that the emotional intervals were judged longer than the sample intervals, *E* = 52.28, ES = 24.35, 95% CI (4.52, 100.04), ddl = 2113.49, *t* = 2.15, *p* = 0.03. Obviously, the same analysis conducted for the control group showed no significant difference between these two intervals, *E* = −24.47, ES = 32.02, 95% CI (−87.26, 38.31), ddl = 2013, *t* = −0.76, *p* = 0.45. A second analysis was conducted to assess the weight of the estimate of the emotional interval on the current reproduction for the following sample intervals (current interval order: N + 1, N + 2, N + 3, N + 4, N + 5). This analysis showed a systematic effect of prior emotional interval for the different successive intervals, and even for the interval most remote from the “emotion prior” (e.g., N + 5). Indeed, our model with the current interval order and the value of the reproduced time for the immediate previous emotional interval as factors showed a significant main effect of the time estimate of emotional interval, *E* = 0.13, ES = 0.05, 95% CI (0.039, 0.2299), ddl = 1411.08, *t* = 2.76, *p* = 0.006, but no main effect of interval order (N + 1, N + 2, N + 3, N + 4, N + 5), *E* = 12.26, ES = 16.0, 95% CI (−19.27, 43.79), ddl = 1372.04, *t* = 0.76, *p* = 0.45, or of the emotion × interval-order interaction, *E* = −0.145, ES = 0.015, 95% CI (−0.044, 0,016), ddl = 1373,13, *t* = −0.94, *p* = 0.35. Therefore, the lengthening of time during the emotional interval led to a constant lengthening of time for the other sample intervals, even those far away from the prior emotional interval. This suggests that the weight (additive) of time reproduced for all sample intervals by the emotion prior did not decrease with the distance from this. For the control group, no effect of the time estimate for the prior emotional interval, *E* = 0.059, ES = 0.107, 95% CI (−0.15, 0.27), ddl = 1359.55, *t* = 0.55, *p* = 0.58, of interval order, *E* = −16.61, ES = 27.91, 95% CI (−71.38, 38.15), ddl = 1323.42, *t* = −0.59, *p* = 0.55, or of the interaction between these factors, *E* = 0.009, ES = 0.033, 95% CI (−0.056, 0.074), ddl = 1323.9, *t* = 0.28, *p* = 0.79, was found.

## Discussion

We tested a new emotion-based version of the ready-set-go procedure used by Jazayeri and Shadlen (2010) to examine the impact of emotional temporal context on current time measurement. With this new version, the results for the control group replicated the finding that the same interval duration is judged shorter when included in a short interval distribution than in a long interval distribution. Therefore, the presence of neutral faces during the sample intervals did not fundamentally change the results since “Vierordt’s law” still held. The originality of our results was to find an overestimation of sample intervals despite there were included in a short interval distribution when a small proportion of intervals (0.16) were emotional (angry faces). For the emotion group, no difference in the judgment of the interval (0.9 s) that overlapped the two temporal distributions was observed when the Short task was performed before the Long task and a reversal effect was observed when the Long task was performed before the Short one, with the overlapping intervals being judged longer in the Short than in the Long task. This demonstrates that not only durations that have just been presented in the same task affect the current time judgment, but that those of another previously performed task also affect this judgment. Durations encountered in the past (and in particular those emotionally charged), therefore constitute reference durations in memory that influence temporal predictions in a new context. In other words, current time judgment is the product of not only the new temporal knowledge in memory but also of older knowledge.

The further analyses of time judgment in the emotion condition (Short task, emotion group) indicate that the intervals were judged longer with the angry face than with the neutral face. This is entirely consistent with the now well-established results on the time-lengthening effect produced by high-arousal negative emotional stimuli (e.g., Gil and Droit-Volet, 2012; Fayolle et al., 2015; Droit-Volet, 2019; Ogden et al., 2019; Piovesan et al., 2019). Nevertheless, the aim of our study was to test the effect of the prior emotional interval in memory on the present time judgment, but not the extension of the emotional reaction triggered in the emotional interval beyond this interval, i.e., on the encoding of subsequent sample intervals. Our results showed the significant impact of the prior time estimate for the emotional interval on the reproduced time for the subsequent sample intervals, regardless of their distance from the emotional interval. One assumption might be that this lengthening effect of estimates for the sample intervals is caused by the emotion induced during the emotional interval, which then persisted beyond this interval. This is, however, not credible because the sample intervals could occur a minute or more after the emotional interval depending on their location in the trial block (e.g., N + 5 with 5 inter-trial intervals). Besides, the time course of the emotional reaction to a picture of a face expressing anger presented on a computer is limited to a short period of time (Droit-Volet, 2019). This observation is consistent with the automatic processing of emotional signals, particularly in the case of emotional faces (Hsiao and Cottrell, 2008; Tracy and Robins, 2008). For instance, some studies have shown that the emotion-related temporal effect does not last long (<1–2 s) in the case of short emotional stimulus presentation on a computer (Angrilli et al., 1997; Noulhiane et al., 2007; Ogden et al., 2019; Piovesan et al., 2019). Moreover, we took the further methodological precaution of incorporating an interval of 4–6 s between two successive trials. Furthermore, in this case, we should have observed a decrease in time estimates with increasing distance between the sample interval and the emotional interval. No such decrease was observed in our study. Another explanation would be that the participants’ expectation of the forthcoming emotional stimulus produced an increase in their arousal level, thus resulting in a lengthening of the estimated duration of the sample intervals. However, this hypothesis is also not very credible, since no increase in time estimates was observed with increasing distance between the emotional interval and the sample interval. Indeed, the longer the time that elapsed, the greater the likelihood of seeing the next emotional stimulus.

Rather than these hypotheses related to an extension of the emotional state beyond the sample intervals, our data provide support for a memory-based hypothesis of the role of reference durations in memory in current time judgments. In line with this assumption, for the short sample intervals, our linear regression analyses indicated differences in the intercept rather than the slope of the time curves between the emotion and control groups. This suggests a general bias in temporal judgment related to a shift in the reference temporal distribution in memory due to the overestimation of sample intervals associated to emotional stimuli. Most models of timing, and even the internal clock models, describe the key role of reference time memory in the present judgment of time (e.g., Gibbon et al., 1984). This has been widely investigated, for example in studies using stimuli of different sensory modalities (auditory, visual) in the same task or in two successive tasks (Penney, 2003). It is therefore both simple and logical to assume that some longer sample durations, those associated with emotional events, were added to the distribution of sample intervals in memory. This would have shifted the mean of the temporal distribution toward a longer value. Consequently, the overlapping interval was judged longer in the short task by the emotion group than by the control group, and this in turn reduced the difference between the time estimates of the overlapping interval in the short and long task or even reversed the effect. Our data therefore provide additional evidence on the key role of previous experience (prior) on perceived intervals (likelihood). In other words, time judgment is not simply the result of an interval measured by an internal clock system, but also of participants’ dispositions based on their knowledge, which is itself updated by experience of stimulus processing (Zhu et al., 2021).

However, it is well established in the literature that memories of threatening events are those that are remembered and recalled best (Ledoux, 1997; Reisberg and Heuer, 2004). Cocenas-Silva et al.’s (2013) study showed that emotional durations associated with threatening stimuli were those that were best recalled from long-term memory. It is therefore likely that durations associated with emotional events do not have the same weight in temporal memory as other durations associated with neutral events. This needs to be tested using our new emotional paradigm in further studies. However, the present study shows a limitation, such as a condition in which both duration ranges (Short and Long) are subject to the introduction of emotional stimuli, or a condition in which no modification of the basic paradigm is performed for direct comparison. Nevertheless, the originality of the present study lies in the development of an emotion-based version of Jazayeri and Shadlen’s procedure and in showing that introducing longer time estimates produced by the perception of emotional events (angry face) in a temporal reproduction task modified the judgment of current intervals by changing the reference temporal distribution in memory. However, a new procedure also raises new questions that must be examined experimentally to better understand the role of emotional priors in the current time judgment. It is clear that this study offers a first step, a test of a new procedure that must be embraced by researchers for the future in the time-emotion domain.

## Data Availability Statement

The original contributions presented in the study are included in the article/supplementary material, further inquiries can be directed to the corresponding author.

## Ethics Statement

The studies involving human participants were reviewed and approved by the UCA Research Ethics Committee (IRB00011540-2019-32). The participants provided their written informed consent to participate in this study.

## Author Contributions

SD-V and SG conceived and planned the design. SD-V conceived the implementation of the research, carried out the analyses of the results and carried out the first draft of the manuscript. SD-V and SG contributed to the final version of the manuscript. Both authors contributed to the article and approved the submitted version.

## Conflict of Interest

The authors declare that the research was conducted in the absence of any commercial or financial relationships that could be construed as a potential conflict of interest.

## Publisher’s Note

All claims expressed in this article are solely those of the authors and do not necessarily represent those of their affiliated organizations, or those of the publisher, the editors and the reviewers. Any product that may be evaluated in this article, or claim that may be made by its manufacturer, is not guaranteed or endorsed by the publisher.
